# Single‐cell and spatial transcriptomics reveal the fibrosis‐related immune landscape of biliary atresia

**DOI:** 10.1002/ctm2.1070

**Published:** 2022-11-04

**Authors:** Chunjing Ye, Jiajie Zhu, Junfeng Wang, Deqian Chen, Lingdu Meng, Yong Zhan, Ran Yang, Shiwei He, Zifeng Li, Shuyang Dai, Yi Li, Song Sun, Zhen Shen, Yanlei Huang, Rui Dong, Gong Chen, Shan Zheng

**Affiliations:** ^1^ Department of Pediatric Surgery Children's Hospital of Fudan University, Shanghai Key Laboratory of Birth Defect, and Key Laboratory of Neonatal Disease Ministry of Health Shanghai China

**Keywords:** biliary atresia, immune, liver fibrosis, single‐cell RNA sequencing, spatial transcriptomics

## Abstract

**Background:**

Biliary atresia (BA) is a devastating inflammatory and fibrosing cholangiopathy of neonates with unknown aetiology. We aim to investigate the relationship between these two main characteristics.

**Methods:**

Single‐cell RNA sequencing and spatial transcriptomics were performed on liver samples from a cohort of 14 objects (BA: *n* = 6; control: *n* = 8). We conducted data integration and cell‐type annotation based on gene expression profiling. Furthermore, we identified fibrosis‐related immune cells according to their spatial locations, GO and KEGG analysis. Finally, SPOTlight and CIBERSORTx were used to deconvolute ST data and microarray data of the GSE46960 cohorts, respectively.

**Results:**

Immune subpopulations inhabiting the ‘fibrotic niche’ (areas of scarring), comprising ‘intermediate’ CD14^++^CD16^+^ monocytes, scar‐associated macrophages, natural killer T cells, transitional B cells and FCN3^+^ neutrophils were identified. GO and KEGG analyses showed that pathways including ‘positive regulation of smooth muscle cell/fibroblast proliferation’ and ‘positive regulation of/response to VEGFR/VEGF/EGFR/FGF’ were enriched in these cell types. Interactions analysis showed that communication among ‘FGF_FGFR’, ‘RPS19‐C5AR1’, ‘CD74_COPA/MIF/APP’ and ‘TNFRSF1A/B_GRN’ was extensive. Finally, the results of deconvolution for ST data and microarray data validated that the proportions of certain identified fibrosis‐related cell types we identified were increased in BA.

**Discussion:**

Fibrosis is an important feature of BA, in which the immune system plays an important role. Our work reveals the subpopulations of immune cells enriched in the fibrotic niche of BA liver, as well as key related pathways and molecules; some are highlighted for the first time in liver fibrosis. These newly identified interactions might partly explain why the rate of liver fibrosis occurs much faster in BA than in other liver diseases.

**Conclusion:**

Our study revealed the molecular, cellular and spatial immune microenvironment of the fibrotic niche of BA.

## Graphical abstract

- Fibrosis‐related immune subpopulations in BA were identified;
- Pathways including ‘positive regulation of smooth muscle cell/fibroblast proliferation’ and ‘positive regulation of/response to VEGFR/VEGF/EGFR/FGF’ may contribute to liver fibrosis in BA;
- Interactions ‘FGF_FGFR’, ‘RPS19‐C5AR1’, ‘CD74_COPA/MIF/APP’ and ‘TNFRSF1A/B_GRN’ may contribute to liver fibrosis in BA.

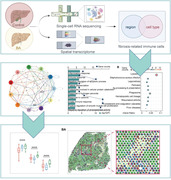

## 1 INTRODUCTION

Biliary atresia (BA) is a devastating inflammatory obliterative cholangiopathy of neonates. It destroys both extrahepatic and intrahepatic bile ducts, disrupts bile flow and produces rapid severe liver fibrosis. Without proper treatment, most BA patients will develop irreversible liver cirrhosis that leads to death in the first 2 years after birth.^1^, ^2^ Though the full aetiological map of BA has not been fully elucidated, some hypotheses suggest that immune factors might be invloved.^3^, ^4^, ^5^, ^6^ Recently, Wang et al. reported a comprehensive description of immune dysfunction (excluding granulocytes) of BA by single‐cell RNA sequencing (scRNA‐seq).^7^ Furthermore, Zhang et al. used CIBERSORTx (https://cibersortx.stanford.edu/) to quantify 22 subsets of immune cells in BA livers and identified eight immune‐related differentially expressed genes (DEGs) and six subtypes of immune cells as critical factors in the progression of the disease.^8^

Liver fibrosis in BA develops faster than any other adult or child liver or biliary disease, and the underlying pathogenic factors of this disease have not been fully elucidated.^9^ Indeed, whether there is a specific relationship between these two main characteristics of BA is of interest. Previously, Ramachandran et al. reported novel scar‐associated macrophages (Mac), endothelial cells and platelet‐derived growth factor receptor‐alpha (PDGFRα) + collagen‐producing mesenchymal cells in cirrhotic human livers by applying scRNA profiling. They identified these subtypes according to their spatial location: the ‘fibrotic niche’ (areas of scarring).^10^ Thus, we speculated that immune cells inhabiting the ‘fibrotic niche’ of BA patients might contribute to liver fibrosis. Here, we combined two new research methods (scRNA‐seq and spatial transcriptomics [ST]) to reveal the cellular, molecular and spatial immune microenvironment of the fibrotic niche of BA livers, aiming to characterize the currently unknown cell types, cell–cell interactions, and key molecules.

## 2 METHODS

### 2.1 Patients, ethics and consent for publication

The study involved 14 subjects at the Children's Hospital of Fudan University, including six patients with type III BA (this type accounts for about 95% of BA) and eight controls (four post‐chemotherapy patients with hepatoblastoma and four with choledochal cyst but no liver damage) during June 2018 to December 2020 (Table S1). BA was diagnosed when the extrahepatic bile duct and/or intrahepatic biliary tree could not be observed by intraoperative cholangiography. The study was approved by the ethics committee of the hospital and informed consent was obtained from each patient's legal guardians.

### 2.2 Tissue dissociation

Livers of patients from BA and control groups were collected during surgery or biopsy and prepared for single‐cell isolation immediately after collection. The tissue was digested enzymatically with DNase I (Sigma‐Aldrich Germany) and collagenase IV (Gibco, USA) at 37°C for 30 min with agitation after being cut into fragments. A 70‐μm cell strainer was used to filter the sample after digestion, and then the tissue was washed with PBS, and centrifuged at 400 × *g* for 5 min. To remove red blood cells and debris from the suspension, Lympholyte‐H separation (Cedarlane, Canada) was used according to the manufacturer's instructions. The pelleted cells were finally resuspended in Dulbecco's Modified Eagle's medium (supplemented with 10% bovine serum albumin) and then assessed with scRNA‐seq analysis.

### 2.3 scRNA‐seq and data processing

Chromium Single‐Cell v.2 equipment (10 x Genomics, USA) was used to perform scRNA‐seq according to manufacturer's instructions. The Chromium Controller was used to generate single‐cell gel bead‐in‐emulsions for cell barcoding. The library was combined and a NovaSeq 6000 (Illumina, USA) (a depth of 400 × 10^6^ reads) software was used for sequence subsequently, raw sequenced data were then converted to FASTQ files with Illumina ‘bcl2fastq’ (v.2.19.1), human genome reference sequence (GRCH38) was used for annotation. After sample demultiplexing, barcode processing and single cell 3′ gene counting using the Cell Ranger (10x Genomics, v.2.1.1) analysis pipeline, a digital gene‐cell matrix was acquired from these data and barcodes with less than 10% of the 99th percentile of total unique molecular identifier (UMI) counts per barcode was filtered.

### 2.4 Quality control, cluster annotation and data integration

For data normalization, dimensionality reduction and clustering, the Seurat v.4.0.0 (https://github.com/satijalab/seurat) in R v.4.1.0 was used. Cells with at least 500 genes detected were considered quantified, with the mitochondrial gene expression percentage to be under 10%. Mitochondrial/ribosomal‐related genes were removed, and the quantified genes were defined as to present (UMI count >0) in at least three cells. DoubletFinder was performed to identify doublets. Data integration was performed using the *Harmony* function. Overall, 109 999 cells remained after quality control. Cell clusters were annotated according to their canonical lineage markers and the singleR package (HumanPrimaryCellAtlasData dataset). The functions FindMakers and FindAllMakers were used to identify DEGs. All subsequent analyses were based on the integrated data.

### 2.5 Cell function analysis and ligand–receptor interaction analysis

Gene ontology (GO) and Kyoto Encyclopedia of Genes and Genomes (KEGG) pathway analyses of DEGs in different clusters were performed in the Database for Annotation, Visualization and Integrated Discovery (DAVID). GO and KEGG terms with a false discovery rate (FDR) of less than 0.05 were considered significantly enriched. Ligand–receptor interaction analysis of scRNA seq data was performed using the iTalk R package, and the Giotto^11^ was performed to identify interactions of ST data.

### 2.6 Spatial transcriptomics

Tissue samples were cut into 10‐μm sections after embedding in optimal cutting temperature compound, A Visium Spatial Gene Expression Kit (10x Genomics) was used for STs. First, the permeability conditions of liver tissue were optimized using the kit for 12 min, and then the sections were stained with haematoxylin and eosin (H&E). After imaging with a Leica DM6000 microscope at a magnification of 20× with the objective lens, ST processing was then performed. The generated complementary DNA libraries were subjected to quality inspection and sequencing using an Illumina Novaseq 6000 system. The loop conditions were set to 28, 98 and 8 for read 1, read 2 and read 3 (i7 index), respectively. The Loupe v.4.0.0 software (10x Genomics) was used by a professional liver pathologist for spots annotation.

### 2.7 Visium spatial transcriptomics data processing

Reads were demultiplexed using Space Ranger software v.1.0.0 (10x Genomics) and annotated with the reference genome GRCh38. Subsequently, the generated count matrices were loaded into Seurat v.4.0.0 environment, and then the data were normalized (using the ‘SCTransform’ function in Seurat for independent tissue sections), reduced and visualized.

### 2.8 Deconvolution analysis

SPOTlight analysis was performed for deconvolution analysis as Elosua‐Bayes et al. reported.^12^ In brief, the proportion of signature of the selected scRNA‐seq cell type is equal to the sum of the proportions of each cell type in different regions, divided by the sum of the proportions of that cell type in all spots. Because of the large number of subgroups, we only defined fibrosis‐related subgroups, and the rest were uniformly defined as another subgroup. For example, monocytes (Mo) was only defined as ‘intMo’ and ‘other Mo’.

### 2.9 Integrated analysis of scRNA‐seq data and microarray data

CIBERSORTx was used to infer the proportion of each fibrosis‐related subtype in patients with BA.^13^ The GSE46960 cohorts data microarray was downloded,^14^ which contained 64 patients with BA, 14 age‐matched subjects with intrahepatic cholestasis as diseased controls and seven healthy subjects.

### 2.10 Statistics

Statistical analysis was performed in R (version 4.0.5). Tests used for statistical significance evaluation are described in detail in the figure legends. All *p*‐values reported are two‐sided.

## 3 RESULTS

### 3.1 Single‐cell landscapes of livers from infants in the BA and control groups

**FIGURE 1:**
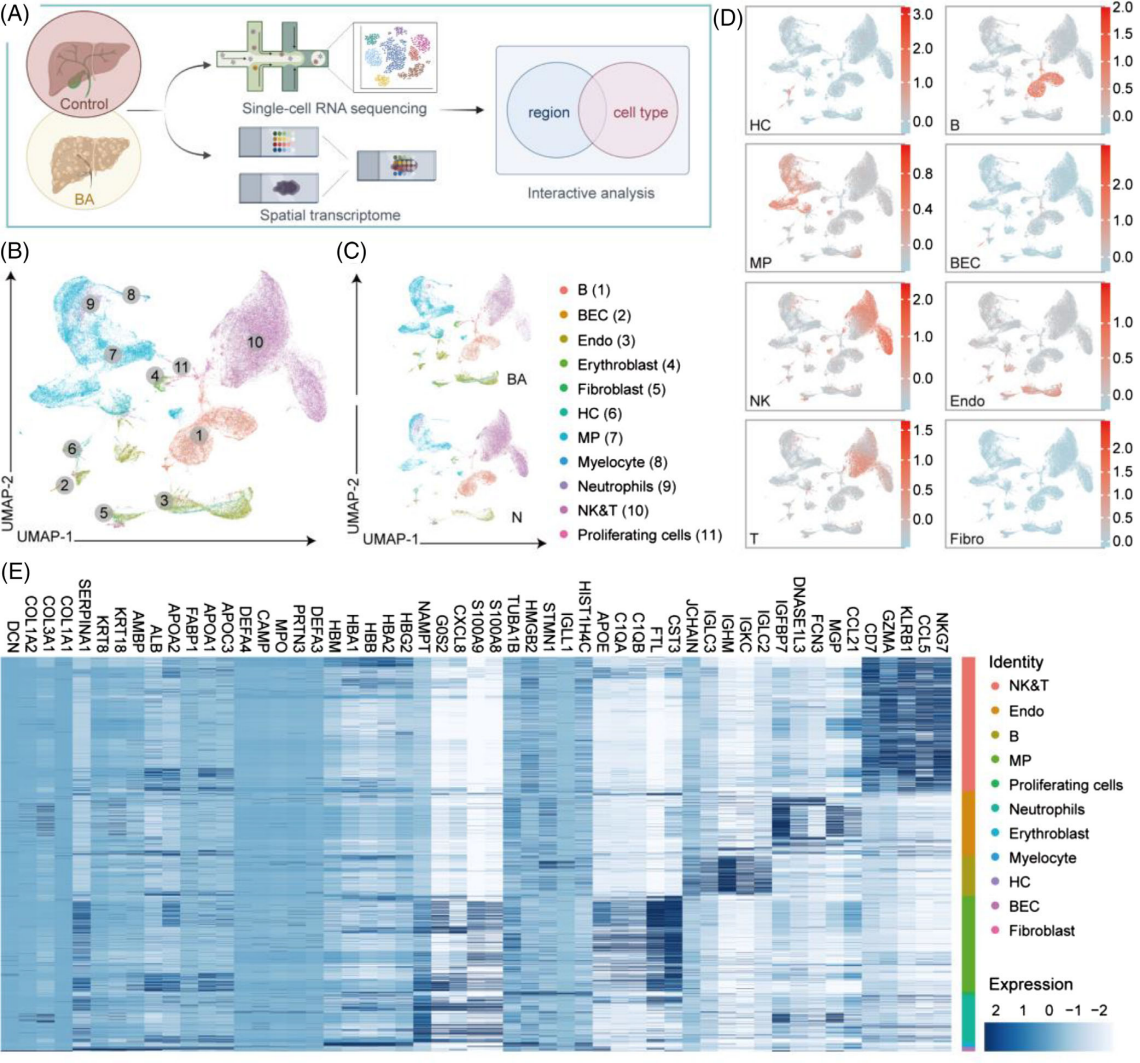
Single‐cell atlas of BA and control livers. (A) Workflow of the whole study, including sample acquisition, sequencing and integration analysis. We obtained the liver biopsies (from six patients with BA and eight normal controls) and performed scRNA‐seq or spatial transcriptome to describe their transcriptional and spatial features. (B) UMAP visualization of 109 999 cells from integrated BA and control samples coloured according to cluster. UMAP: uniform manifold approximation and projection. (C) UMAP visualization of cells split by sample coloured according to cluster. (D) Featureplots: scores of canonical genes of different lineages. The colour indicates the scaled mean expression of genes. BEC, biliary epithelial cells; HC, hepatocytes; NK, natural killer cells; T, T cells; B, B cells; MP, mononuclear phagocytes; Endo, endothelial cells. (E) Heatmap: expression of the top five DEGs of different lineages. The colour indicates the scaled expression of genes.

To elucidate the cellular architecture of BA, we analyzed three liver biopsies of infants with BA and three adjacent normal liver biopsies of children with hepatoblastomas, using scRNA‐seq (Figure 1A). In total, 109 999 single cells passed quality control (Figure S2), and to reveal 29 populations (Figure 1B), each containing cells from both BA and/or control livers (Figure 1C), 11 main cell lineages were annotated using canonical lineage markers and SingleR (Figure 1D,E; Table S3 ; Figure S4). The 11 main lineages include mononuclear phagocytes (MP), natural killer cells and/or T (NK & T) cells, B cells, neutrophils, myelocytes, erythroblasts, fibroblasts, hepatocytes, bile duct epithelial cells, endothelial cells (Endo) and proliferating cells. All markers refer to published gene signatures. Subpopulation markers were identified across lineages to provide reference for future research (Figure 1E; Table S5).

### 3.2 Spatially resolved heterogeneity of livers from infants in the BA and control groups

To gain insights into the spatial organization of cell types, we performed ST on one BA sample and one control sample (Figure 1A). After filtering data, 3638 high‐quality spots were detected in the BA liver, and each spot contained a mean of 89 199 clean reads, 4937 detected genes and 23 742 UMIs. In the control sample, the liver contained a total pool of 1909 individual spots with a mean of 186 510 reads, 3195 genes and 14 126 UMIs per spot. Because differences in histology exists (e.g., highly fibrotic areas with dense connective tissue contain fewer cells than areas with high cell density in the control liver), we expected these statistics could be variable in BA.

**FIGURE 2:**
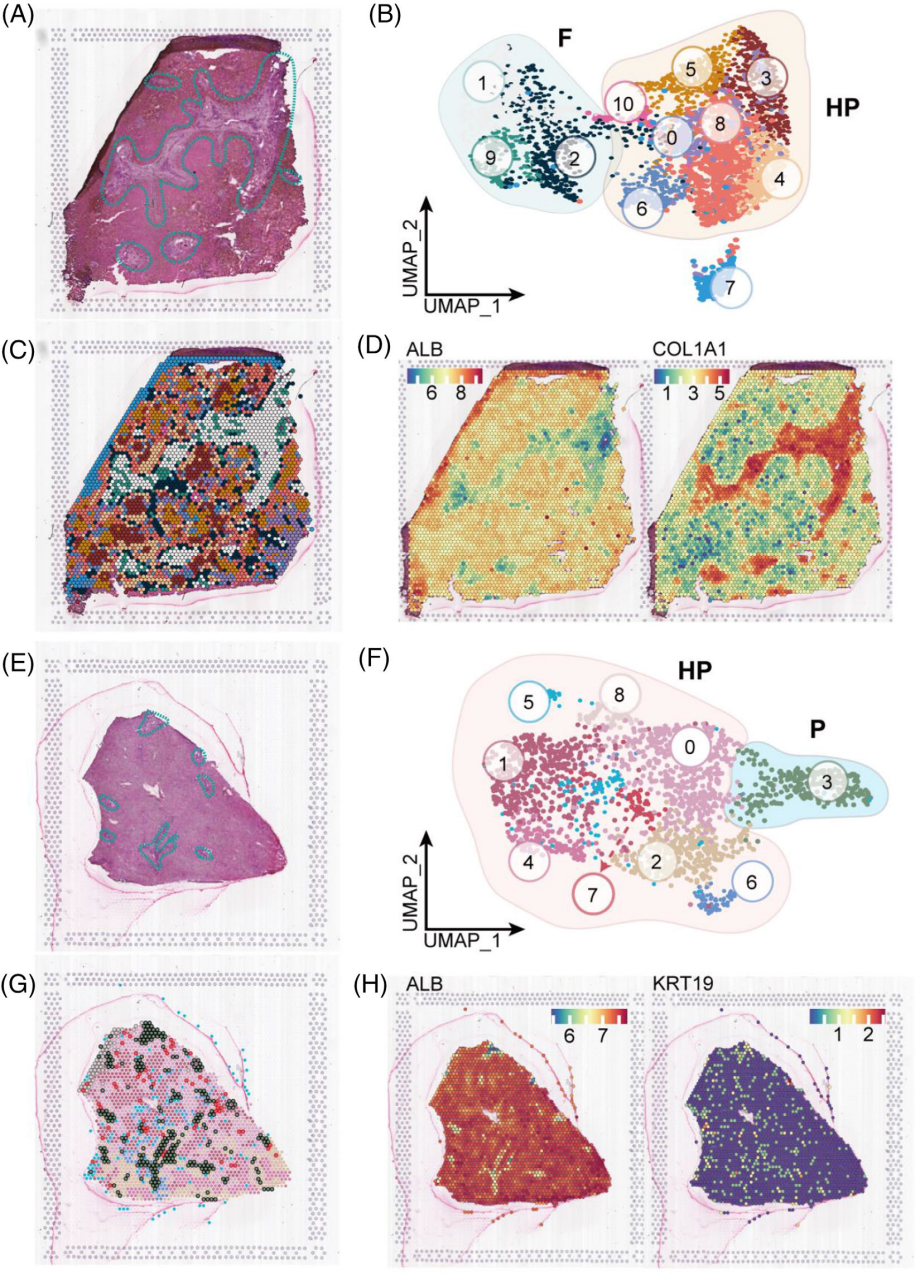
Mapping BA liver heterogeneity using spatially resolved transcriptomics and single cells. (A) H&E staining of different regions of BA liver tissue including the hepatic parenchymal area (HP) and fibrotic niche (F; blue dotted line). (B) UMAP visualization of 11 major clusters of BA liver by ST. The clusters 1, 2 and 9 in the green circle were localized to the fibrotic niche (F); the eight other clusters in orange circles were localized to the HP. (C) The spots are visualized as coloured according to cluster at their original positions in the BA liver. (D) Spatial plots showing the expressions of marker genes for hepatocytes (*ALB*) and fibrosis (*COL1A1)* in BA liver. (E) H&E staining of different regions for control liver including the HP and portal areas (blue dotted line). (F) UMAP visualization of nine major clusters of BA control by ST. The cluster in the blue circle was localized in the portal area (P). The other eight clusters in the orange circles were localized in the HP. (G) The spots are visualized as coloured according to cluster at their original positions in the control liver. (H) Spatial plots showing the expressions of marker genes for hepatocytes (*ALB*) and bile duct epithelial cells (*KRT19*) in control liver

We first annotated slides for distinct histological features after H&E staining and brightfield imaging (Figure 2A,E). Two main regions were defined in BA section: the hepatic parenchymal area (HP) and fibrotic niche (F) (Figure 2A). The control tissue section did not contain a fibrotic niche, so it was defined by the HP and portal area (P) (Figure 2E). Through dimensionality reduction and clustering the spots of each ST array, we identified 11 and 9 major cell clusters in the BA liver (Figure 2B,C) and control liver (Figure 2F,G), respectively, based on gene expression pattern, respectively. Subclusters of two samples were then classified into three regions of HP, F, and P according to their spatial locations (Figure 2C,G), as well as expression key marker genes of hepatocytes (*ALB*), bile duct epithelial cells (*KRT19*) and fibrosis (*COL1A1*) (Figure 2D,H).

### 3.3 Distinct subpopulations of mononuclear phagocytes inhabit the fibrotic niche

**FIGURE 3:**
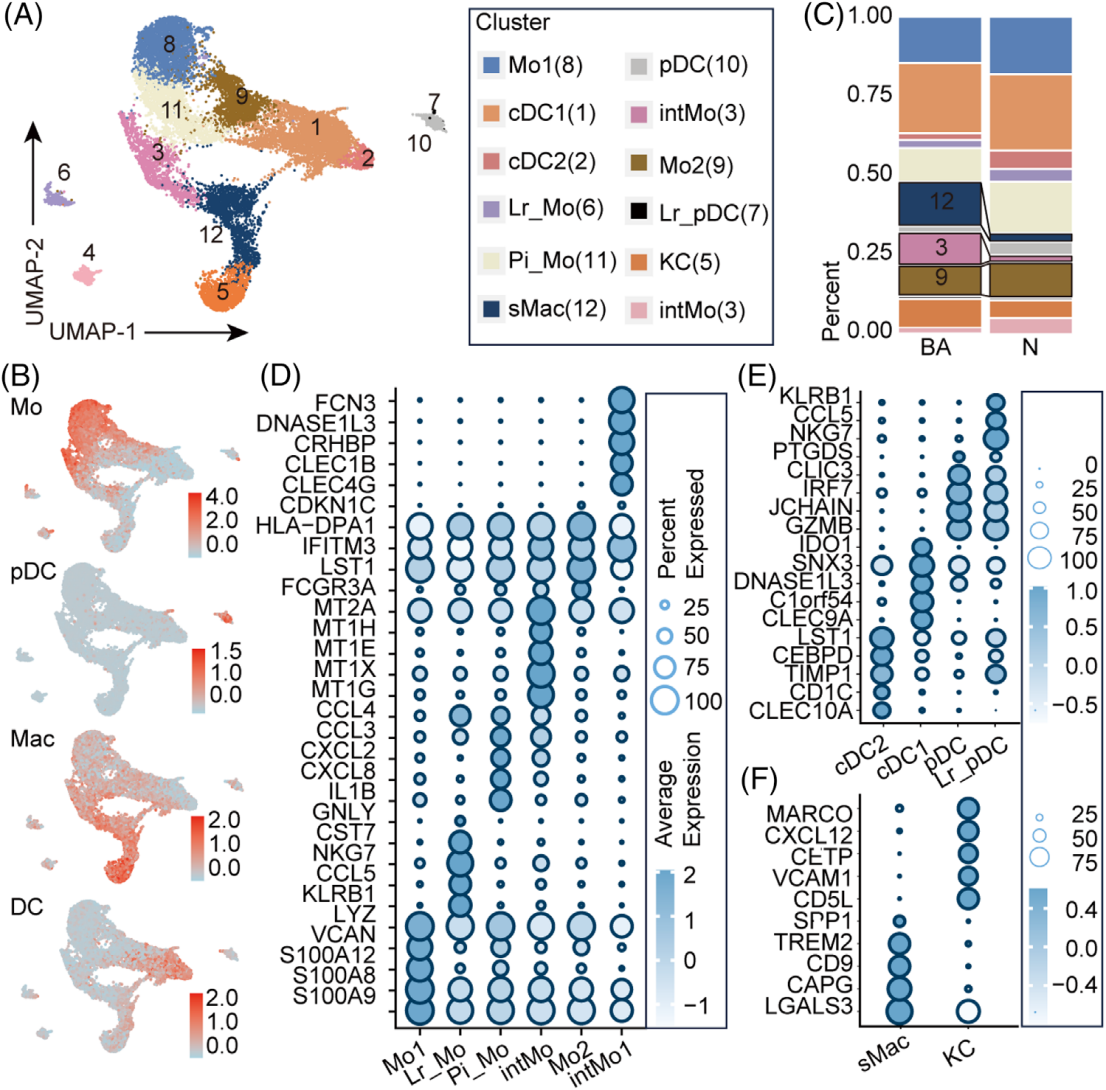
Distinct subpopulations of mononuclear phagocytes (MP) inhabit the fibrotic niche. (A) UMAP visualization of 12 subclusters of MP from integrated BA and control sample data from scRNA‐seq. (B) Feature plots of total scores of marker genes of different lineages. The colour indicates the module scores of marker genes of different lineages. Details of marker genes are shown in Table S2. Mo, monocytes; DC, dendritic cells; Mac, macrophages; pDC, plasmacytoid DCs. (C) Fractions of every subcluster of MP in BA and control livers (*p* < .0001, ****, chi‐square test) (D–F) Heatmaps: expressions of the top five DEGs in subclusters of dendritic cells, monocytes and macrophages from scRNA‐seq data. The colour of the heatmap indicates the scaled mean expression of genes and the colour of the bar above represents the clusters in (A).

Clustering of MPs in scRNA‐seq data identified 12 clusters belonging to three major lineages, including dendritic cells (DCs), Mo and Mac, each identified by their unique signature genes (Figure 3A,B). DCs were annotated as conventional type 1 CLEC9A^+^ DCs (cDC1, cluster 1), CD1C^+^ DCs (cDC2, cluster 2), plasmacytoid DCs (pDC, cluster 10) and liver‐resident plasmacytoid DCs (Lr_pDC, cluster 7), according to their classic markers and DEGs^15^ (Figure 3E). pDC and cDC subtypes play different roles in immune response, and their expression of sensors, pathways and effectors is heterogeneous. While pDCs specialize in producing type I interferon to cope with viral infections, cDCs are mainly effective in antigen‐specific stimulation of T cells.^16^, ^17^, ^18^ Mo were annotated as CD14^+^CD16^−^ Mo (‘classical’, Mo1, cluster 8), CD14^+^CD16^++^ Mo (‘nonclassical’, Mo2, cluster 9), ‘intermediate’ CD14^++^CD16^+^ Mo (intMo, cluster 3, 4), Lr_CD14^++^CD16^+^ Mo (Lr_Mo cluster 6) and ‘pro‐inflammatory’ IL1B^+^Mo (Pi_Mo, cluster 11) according to the expressions of CD14, CD16 and MKI67 (Figure 3D). Mac were annotated as scar‐associated macrophages (sMac, cluster 12) and Küpffer cells (KC, cluster 5) according to their corresponding markers^15^ (Figure 3F) and the top 10 DEGs were identified to better classify cells into these two cell populations in BA tissues. Proportions of these 12 clusters were then estimated from the BA and control data (Appendix S6). For BA and controls, CD14^+^CD16^++^ Mo (‘nonclassical’, Mo2, cluster 9) accounted for 9.74% and 11.38%; liver‐resident plasmacytoid DCs (Lr_pDC, cluster 7) accounted for 0.24% and 0.07%; ‘intermediate’ CD14^++^CD16^+^ Mo (cluster 3) accounted for 10.58% and 1.67%; and sMac accounted for 14.62% and 2.37%, respectively (Figure 3C; *p* < .0001).

**FIGURE 4:**
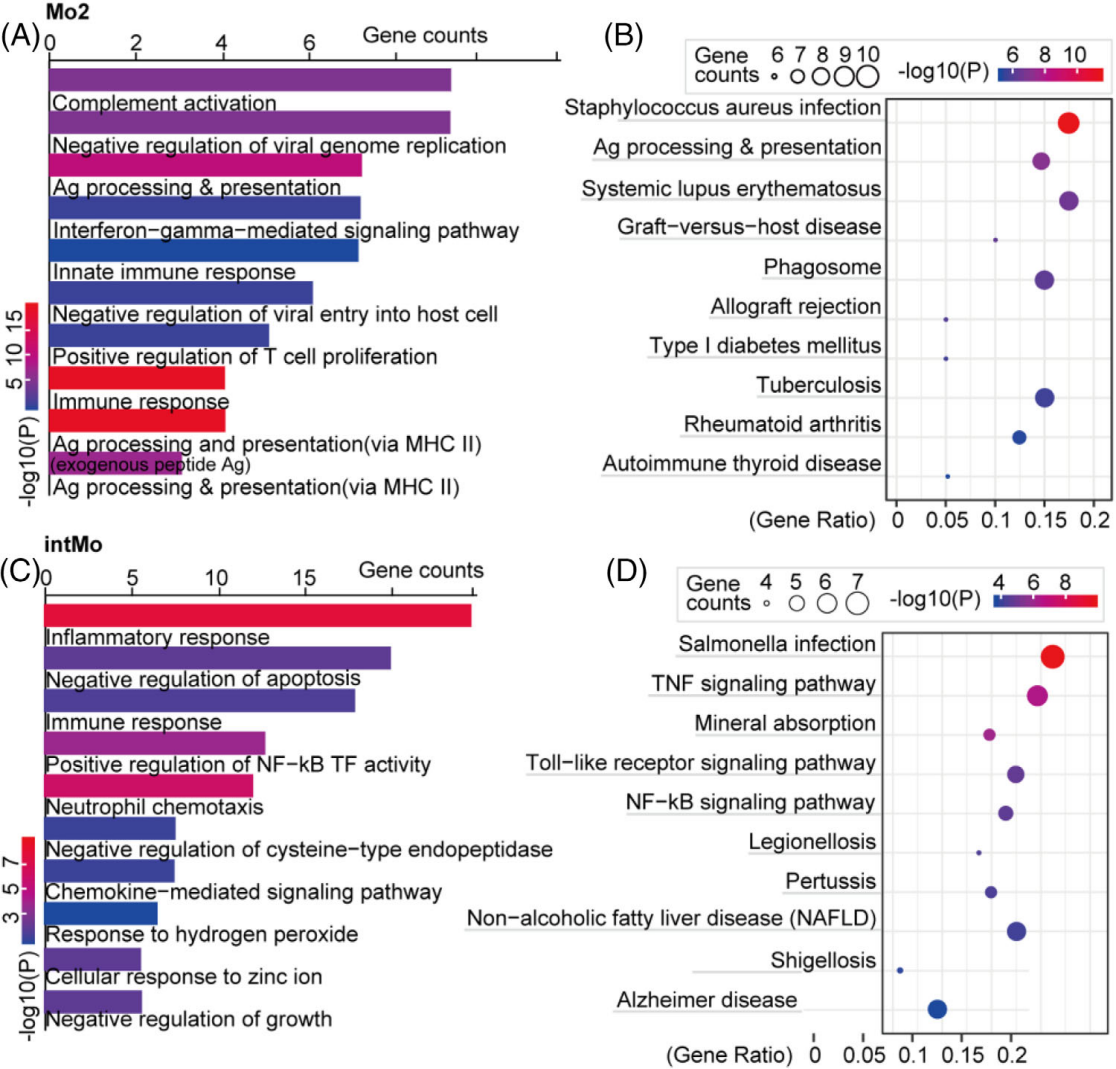
Gene ontology (GO) analysis of two subclusters of monocytes with increased fractions in BA from scRNA‐seq data. (A) Analysis of BP based on DEGs of CD14^+^CD16^++^ Mo (‘nonclassical’, Mo2, cluster 9) from scRNA‐seq data. The colour indicates the log10(*P*) of each term and the bar indicates the gene counts. (B) Analysis of KEGG pathways based on DEGs of CD14^+^CD16^++^ Mo (‘nonclassical’, Mo2, cluster 9) from scRNA‐seq data. The bar indicates the log10(*P*) of each term and the colour indicates the gene counts. (C) Analysis of biological process (BP) based on DEGs of ‘intermediate’ CD14^++^CD16^+^ Mo (cluster 3) from scRNA‐seq data. The colour indicates the log10(*P*) of each term and the bar indicates the gene counts. (D) Analysis of KEGG pathways based on DEGs of ‘intermediate’ CD14^++^CD16^+^ Mo (cluster 3) from scRNA‐seq data. The colour indicates the log10(*P*) of each term and the bar indicates the gene counts.

**FIGURE 5:**
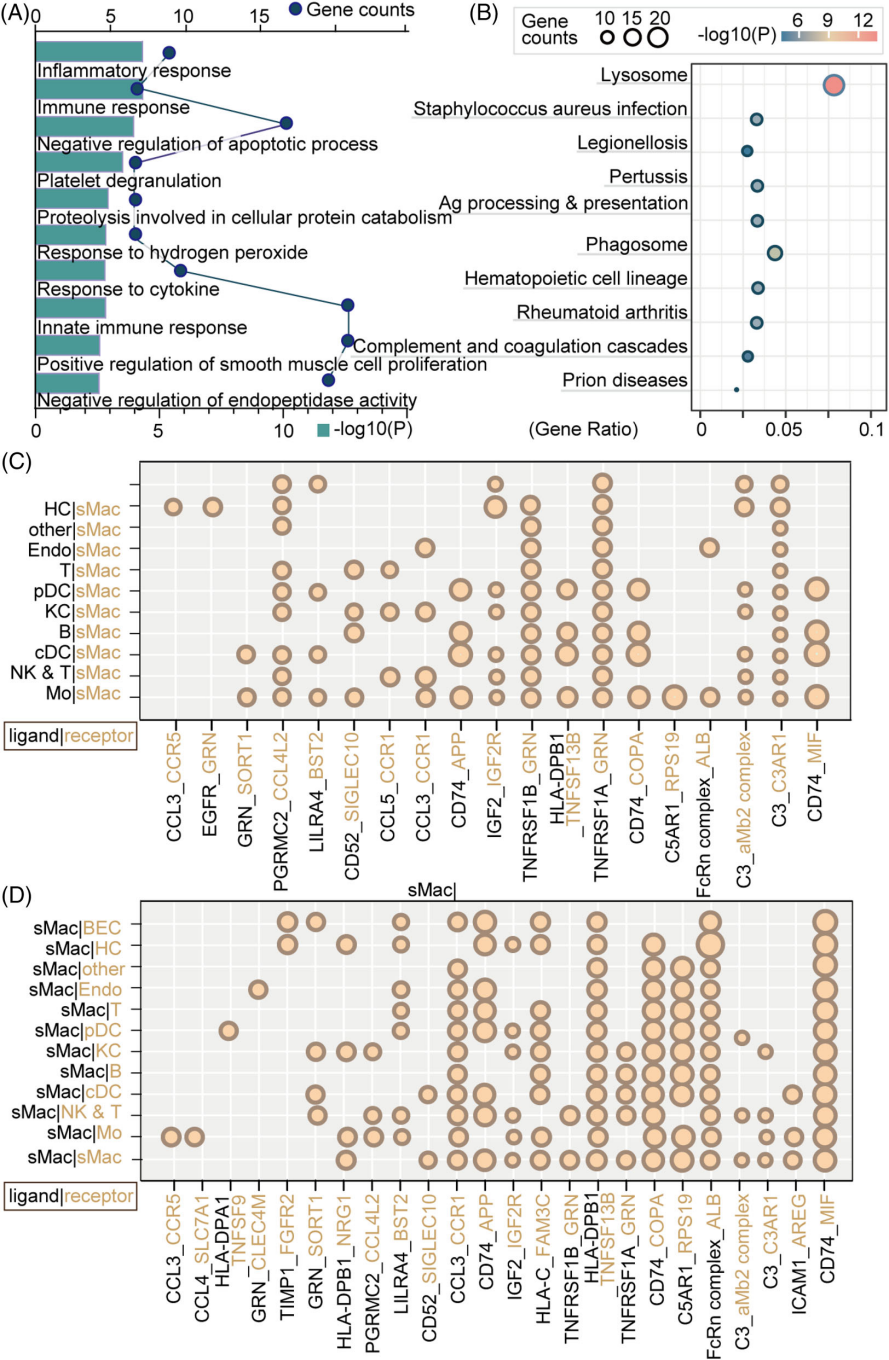
GO analysis of sMac. (A) Analysis of biological process (BP) based on DEGs of sMac from scRNA‐seq data. The bar indicates the log10(*P*) of each term and the circle indicates the gene counts. (B) Analysis of KEGG pathways based on DEGs of sMac from scRNA‐seq data. The colour indicates the log10(*P*) of each term and the size of the circle indicates the gene counts. (C and D) Ligand–receptor interaction analysis between sMac and other cell types. Black font indicates ligands and yellow font indicates receptors. The proportion of interactions is represented by the size of the circle.

To further characterize the functions of subgroups with increased proportions in BA, we performed GO enrichment analysis of these clusters. The results indicated enrichment of many immune process terms, including ‘neutrophil chemotaxis’, ‘immune response’, ‘inflammatory response’, ‘complement activation’, ‘TNF signaling pathway’ and ‘NF‐κB signaling pathway’, in intMo (clusters 3; Appendix S7). Biological processes (BP) ‘antigen processing and presentation’ and ‘negative regulation of viral genome replication and viral entry into host cell’ were enriched in CD14^+^CD16^++^ Mo (‘nonclassical’, Mo2, cluster 9) (Figure 4; Appendix S7). To delineate the functional profile of sMac, we performed GO enrichment analysis and unbiased ligand–receptor interaction analysis. In addition to immune and inflammatory responses, fibrosis ‘positive regulation of smooth muscle cell (SMC) proliferation’ was also enriched. Interactions such as ‘CCL5/3_CCR1/5’, ‘CD74_COPA/MIF/APP’ and ‘TNFRSF1A/B_GRN’ showed high activity, which was mostly consistent with previous research (Figure 5; Appendix S7).^10^, ^15^

**FIGURE 6:**
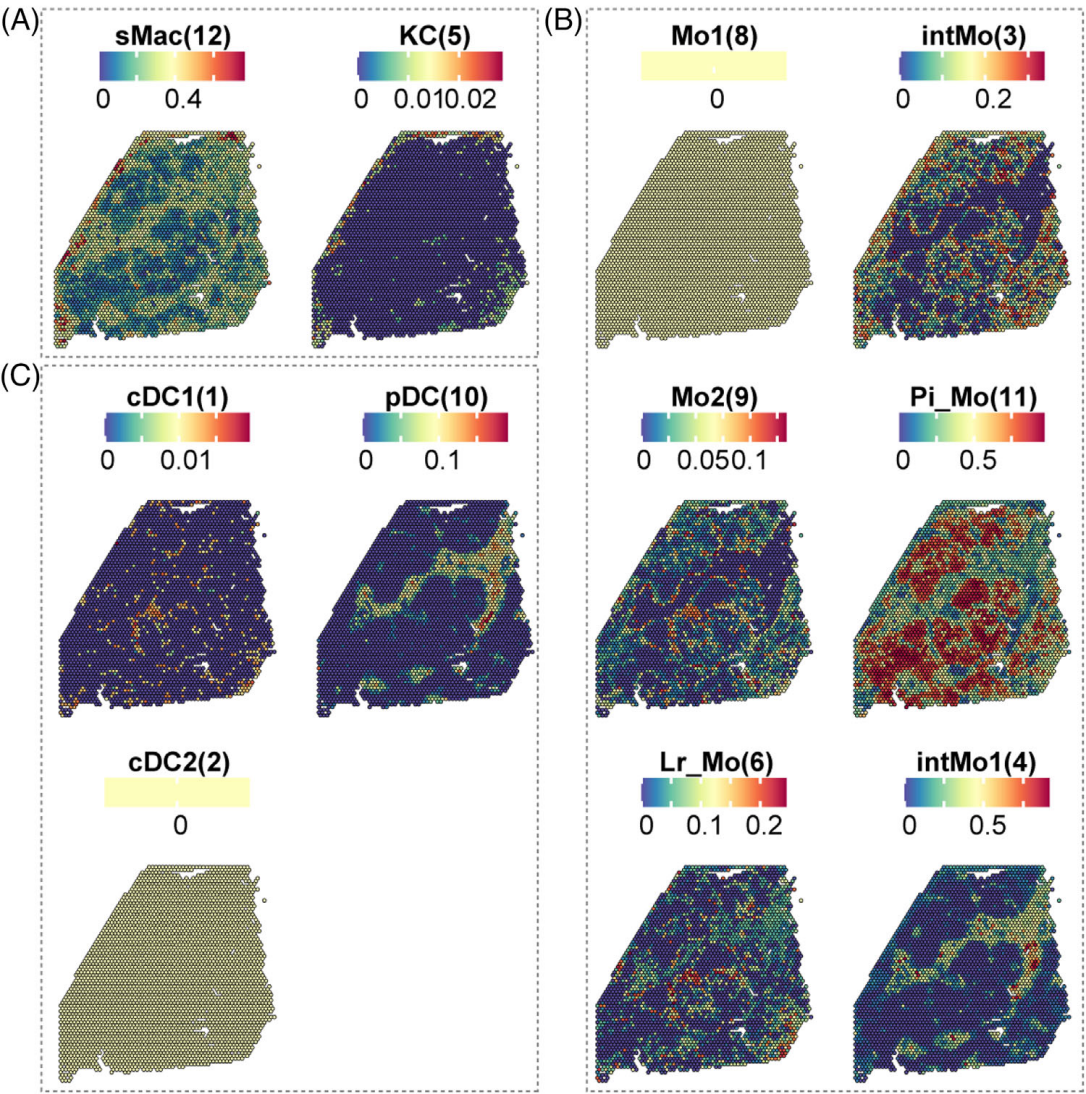
Visualization of MP subpopulations by ST. (A) Spatial expression of sMac and KC from scRNA‐seq data. The colour indicates the expression levels of the subgroup genes. (B) Spatial expression of Mo subclusters from scRNA‐seq data. The colour indicates the expression levels of the subgroup genes. (C) Spatial expression of DC subclusters. The colour indicates the expression levels of the subgroup genes.

The visualization of MP subpopulations in ST data of BA showed that sMac (cluster 12) was clustered in areas of liver fibrosis, which confirms that this subgroup is closely related to liver fibrosis in BA (Figure 6A). In addition, pDC (cluster 10) and intMo (cluster 3) were present in the fibrotic niche (Figure 6B,C). Further GO enrichment analysis showed that pDC had several BPs involved in ‘positive regulation of SMC and fibroblast proliferation’ and ‘positive regulation of/response to vascular endothelial growth factor receptor (VEGFR)/vascular endothelial growth factor (VEGF)’, which promote liver fibrosis (Appendix S7).^19^, ^20^ The ‘intermediate’ CD14^++^CD16^+^ Mo also displayed BPs involved in ‘positive regulation of SMC proliferation’ and ‘positive regulation of/response to vascular VEGF/epidermal growth factor receptor (EGFR)/fibroblast growth factor (FGF)’, indicating that these two subpopulations may also promote liver fibrosis.

### 3.4 Distinct NK and T‐cell subpopulations inhabit the fibrotic niche

**FIGURE 7:**
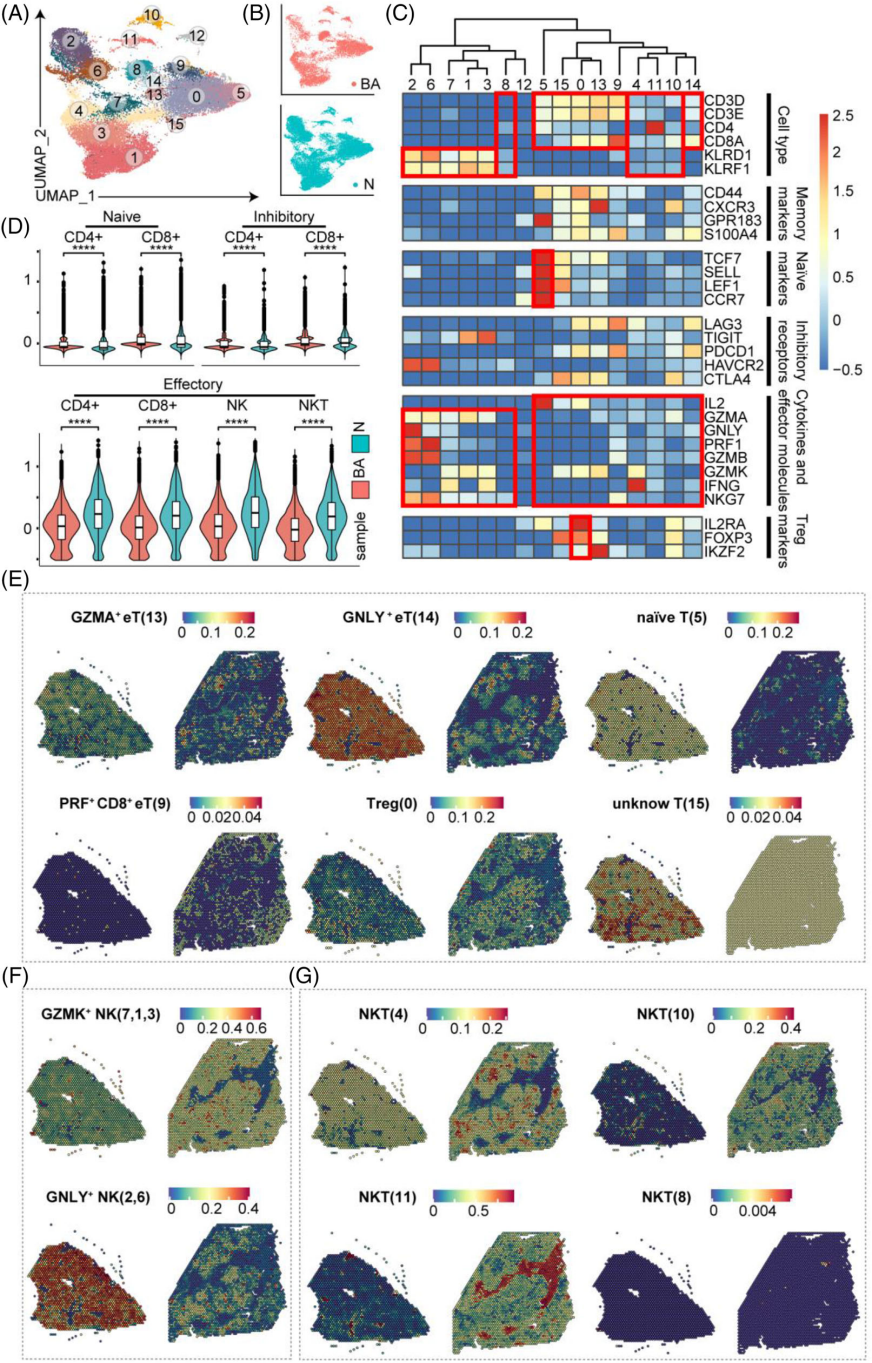
Distinct subpopulations of natural killer and/or T (NK & T) cells inhabit the fibrotic niche. (A) UMAP visualization of NK & T subclusters from scRNA‐seq data. (B) UMAP visualization of cells coloured by samples. (C) Heatmap: scores of normalized mean expressions of selected module genes of NK cells and T cells (including genes related to cell type identification, naiveness and other functional genes) in each cell cluster. Red boxes highlight the prominent patterns defining known NK and T‐cell subtypes. (D) Vlnplots of scores of cell module genes of NK & T subclusters in BA and control livers. The colour indicates the group (*p* < .0001, ****; ns, no significance, *t*‐test). (E–G) Spatial expression of NK & T subclusters in BA and control livers from scRNA‐seq data

To address the NK and T‐cell heterogeneity, the expression of signature genes and known functional markers were used in the annotation.^21^, ^22^ Clustering of the ‘NK & T’ group in scRNA‐seq data identified 15 clusters (Figure 7A,B), annotated as regulatory T cells (Treg, cluster 0), naïve T cells (cluster 5), GZMA^+^ effector T cells (GZMA^+^ eT, cluster 13), unknown T cells (cluster 15), GNLY^+^ NK cells (GNLY^+^ NK, clusters 2 and 6), GZMK^+^ NK cells (GZMK^+^ NK, clusters 1, 3 and 7), GNLY^+^CD8^+^ effector T cells (GNLY^+^CD8^+^ eT, cluster 14), PRF1^+^CD8^+^ effector T cells (PRF1^+^CD8^+^ eT, cluster 9) and NKT cells (clusters 4, 8, 10 and 11) (Figure 7C). To further evaluate the states of NK & T subpopulations in BA and control groups, we defined scores of naivety, exhaustion and cytotoxicity based on previously defined gene signatures.^21^ All scoring levels in the BA group were lower than the control group (Figure 7D). Visualizing the spatial expression of these subclusters demonstrated that most T cells and NK cells had lower expression levels in BA, whereas NKT cells had higher levels compared with controls. Furthermore, cluster 11 was also expanded in cirrhotic livers (Figure 7E).

### 3.5 Distinct B‐cell subpopulations inhabit the fibrotic niche

**FIGURE 8:**
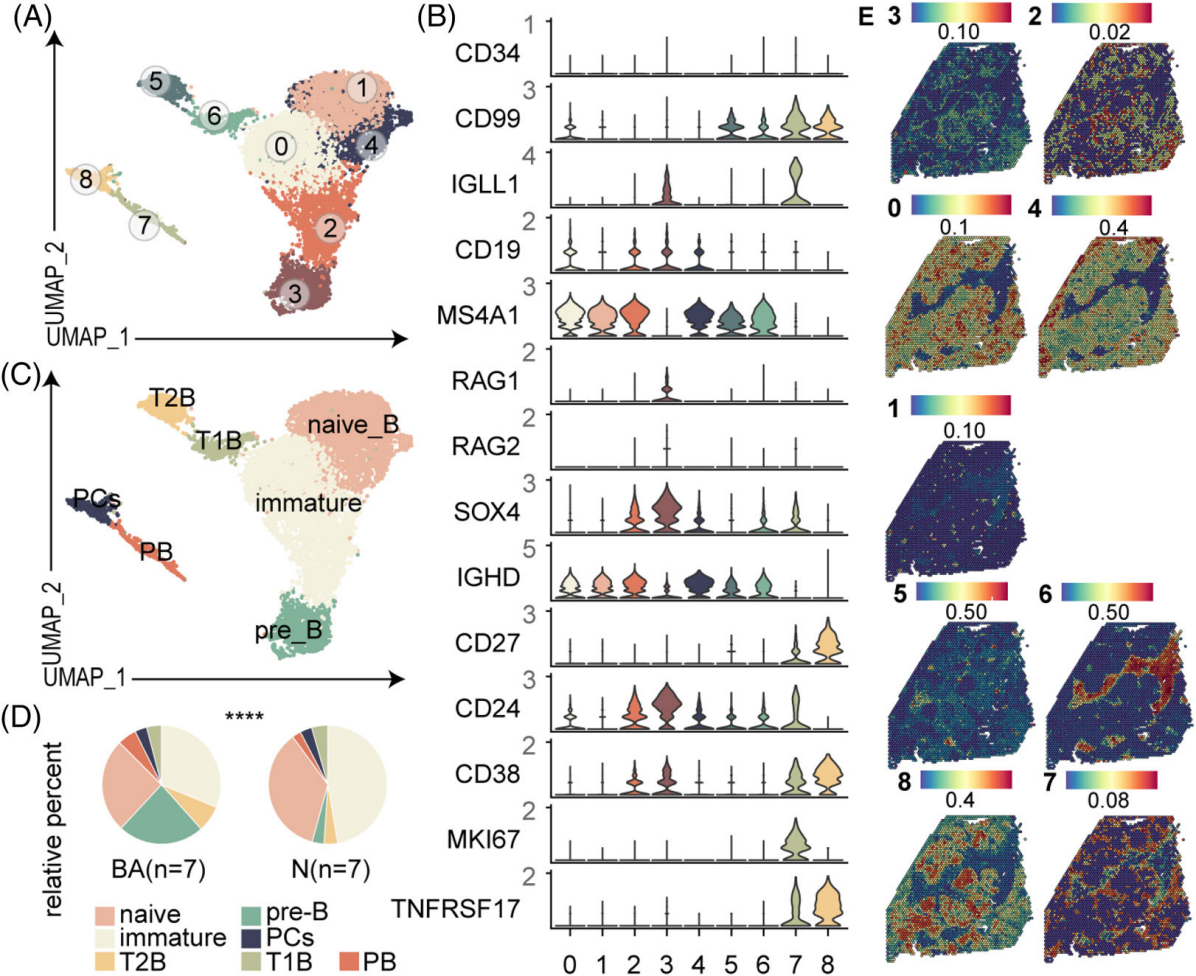
Distinct subpopulations of B cells inhabit the fibrotic niche. (A) UMAP visualization of B subclusters from scRNA‐seq data. (B) Vlnplots: expressions of some marker genes of B subclusters. The colour indicates the group (*p* < .0001, ****; ns, no significance; chi‐square test). (C) UMAP visualization of cell definitions. Naïve, naïve B cells; Immature, immature B cells; TB, transitional B cells; PCs, plasma cells; PB, plasmablast. (D) The fractions of B subclusters in BA and control livers. (E) Spatial expression of B subclusters in BA and control livers from scRNA‐seq data

Wang et al. provided a comprehensive annotation of B cells in cases of BA and discovered that the lymphopoiesis of hepatic B cells does not stop after birth, and in this case, tolerance deficiency leads to accumulation of immunoglobulin G (IgG) autoantibodies.^7^, ^22^ Therefore, we annotated our B cells in our scRNA‐seq data according to their markers and identified nine subclusters belonging to seven subtypes (Figure 8A). These were naïve B cells (clusters 1 and 4), immature B cells (clusters 0 and 2), transitional B cells (T1B: cluster 6 and T2B: cluster 5), plasma cells (PCs, cluster 8) and plasmablasts (PB, cluster 7) (Figure 8B,C). Proportions of these clusters were estimated from the BA and control data (Figure 8D).

Visualizing B subpopulations spatially, we found that T1B (cluster 7) inhabited the fibrotic niche (Figure 8E), and GO enrichment analysis showed that T1B had BPs involved in ‘positive regulation of fibroblast proliferation’ and ‘response to VEGF/FGF’ (Appendix S7), which indicates these two subpopulations may also promote liver fibrosis.

### 3.6 Neutrophils inhabit the fibrotic niche and have multilineage interactomes with other cell lineages

**FIGURE 9:**
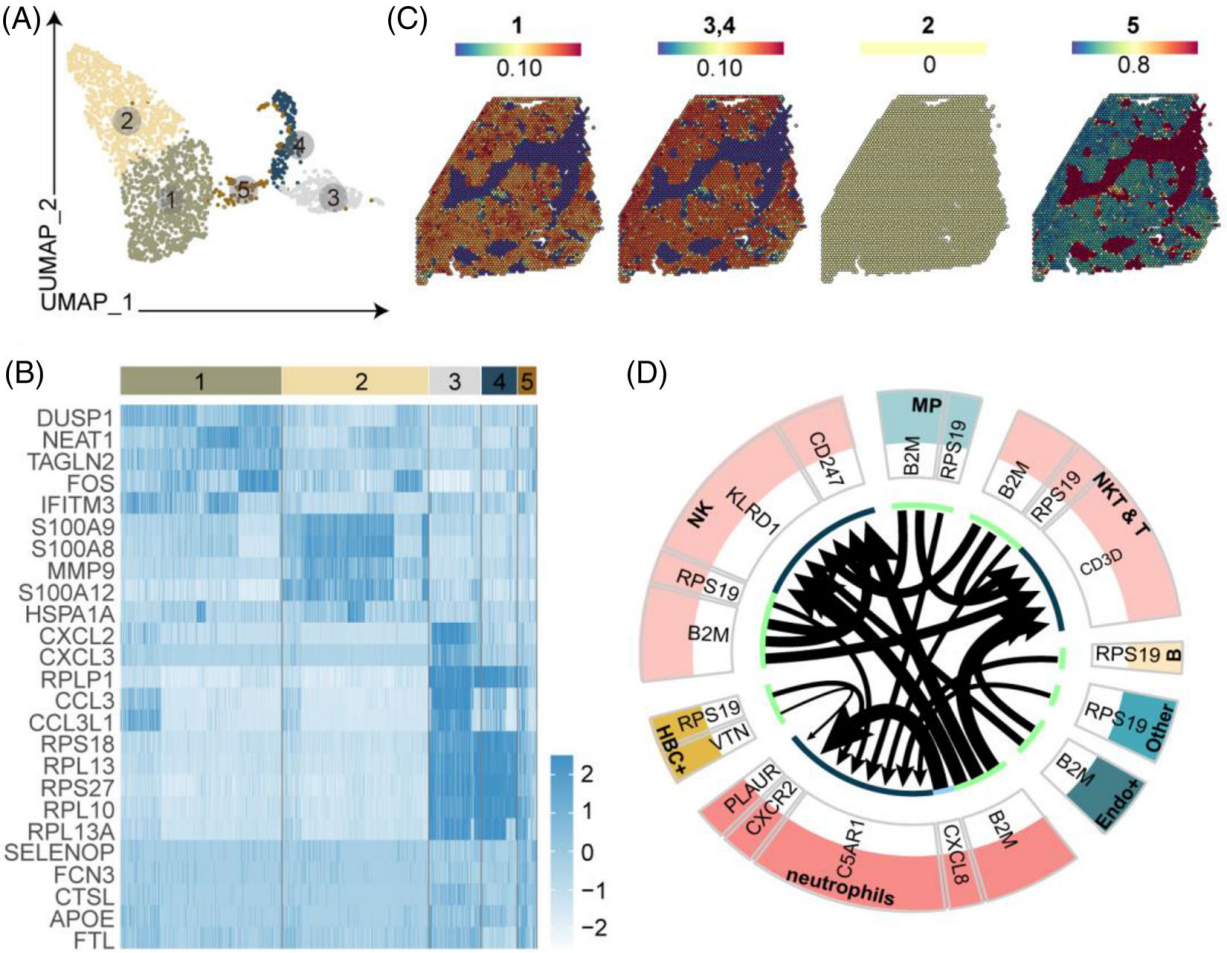
Distinct subpopulations of neutrophils inhabit the fibrotic niche. (A) UMAP visualization of neutrophil subclusters from scRNA‐seq data. (B) Heatmap of the top five DEGs in neutrophil subclusters. The colour of the heatmap indicates the scaled mean expression of genes and the colour of the bar indicates the subclusters. (C) Spatial expression of neutrophil subclusters in BA and control livers from scRNA‐seq data. (D) Ligand–receptor interaction analysis of neutrophils and other cell types. The colour indicates the cell type and the thickness of the arrow represents the strength of the association. The green part of the circle represents the ligand and the black part of the circle represents the receptor.

Given that a variety of immune cells have BP involved in ‘neutrophil chemotaxis’, and that neutrophils have not been studied in depth previously, we conducted a further analysis of these cell types. We identified five subclusters (Figure 9A) in scRNA‐seq data, and the top five DEGs were identified for the optimal grouping of these cells (Figure 9B).

Visualizing neutrophils spatially in BA, we found that FCN3^+^ neutrophils (cluster 5) specifically inhabited the fibrotic niche area (Figure 9C), and GO enrichment analysis showed that this subtype had BPs involved in ‘positive regulation of EGFR’ and ‘FGFR signaling pathway’ (Appendix S7), indicating they may promote liver fibrosis. To further explore the cell‐to‐cell communications with other cell lineages, a ligand–receptor interaction analysis was performed using iTALK.^23^ The interaction ‘RPS19‐C5AR1’ is widely present in the interaction between various immune cells and neutrophils, suggesting it might help neutrophils fulfil their important role (Figure 9D). Interestingly, it was reported that complement factor 5 has a causal role in fibrogenesis across species^24^ and might act via the C5AR1‐Hippo‐YAP pathway.^25^

### 3.7 Key pathways and molecules that promote liver fibrosis

**FIGURE 10:**
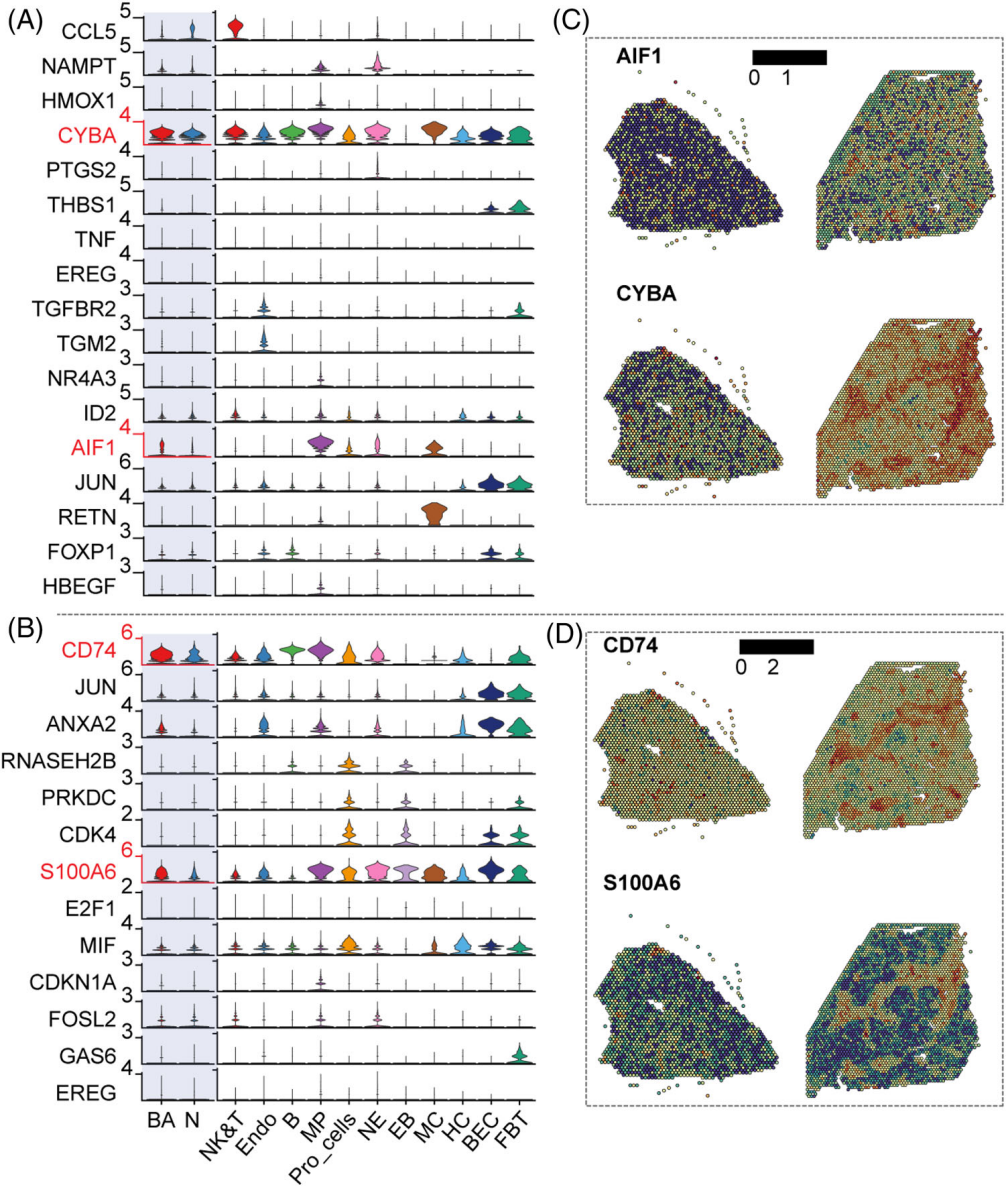
Key pathways and molecules that promote liver fibrosis. (A) Vlnplots: expression of genes involved in ‘positive regulation of SMC and proliferation’ in different samples and cell lineages. The colour indicates the group (*p* < .0001, ****; ns, no significance, *t*‐test). (B) Vlnplots: expression of genes involved in ‘positive regulation of fibroblast proliferation’ in different samples and cell lineages. The colour indicates the group (*p* < .0001, ****; ns, no significance, *t*‐test). NE, neutrophils; EB, erythroblasts; MC, myelocytes; FBT: fibroblasts; Pro_cells, proliferating cells. (C) Spatial expression of AIF1 and CYBA in BA and control livers. (D) Spatial expression of CD74 and S100A6 in BA and control livers

After revealing a comprehensive atlas of immune microenvironment in the fibrotic niche in BA, we finally explored the gene expression and spatial expression of key pathways that promote liver fibrosis, including genes involved in the ‘positive regulation of SMC proliferation’ (*CCL5*, *NAMPT*, *HMOX1*, *CYBA*, *PTGS2*, *THBS1*, *TNF*, *EREG*, *TGFBR2*, *TGM2*, *NR4A3*, *ID2*, *AIF1*, *JUN*, *RETN*, *FOXP1* and *HBEGF*) and ‘positive regulation of fibroblast proliferation’ (*CD74, JUN, S100A6, RNASEH2B*, *PRKDC*, *CDK4*, *S100A6*, *E2F1*, *MIF*, *CDKN1A*, *FOSL2*, *GAS6* and *EREG*), among which the expressions of *CYBA*, *AIF1*, *CD74* and *S100A6* were increased significantly in BA samples compared with those in controls (Figure 10A,B), and were concentrated in the fibrotic niche (Figure 10C,D). This suggests that these genes have a particularly important role in promoting liver fibrosis, which is worthy of further investigation.

### 3.8 Integrated analysis of scRNA data and ST data

**FIGURE 11:**
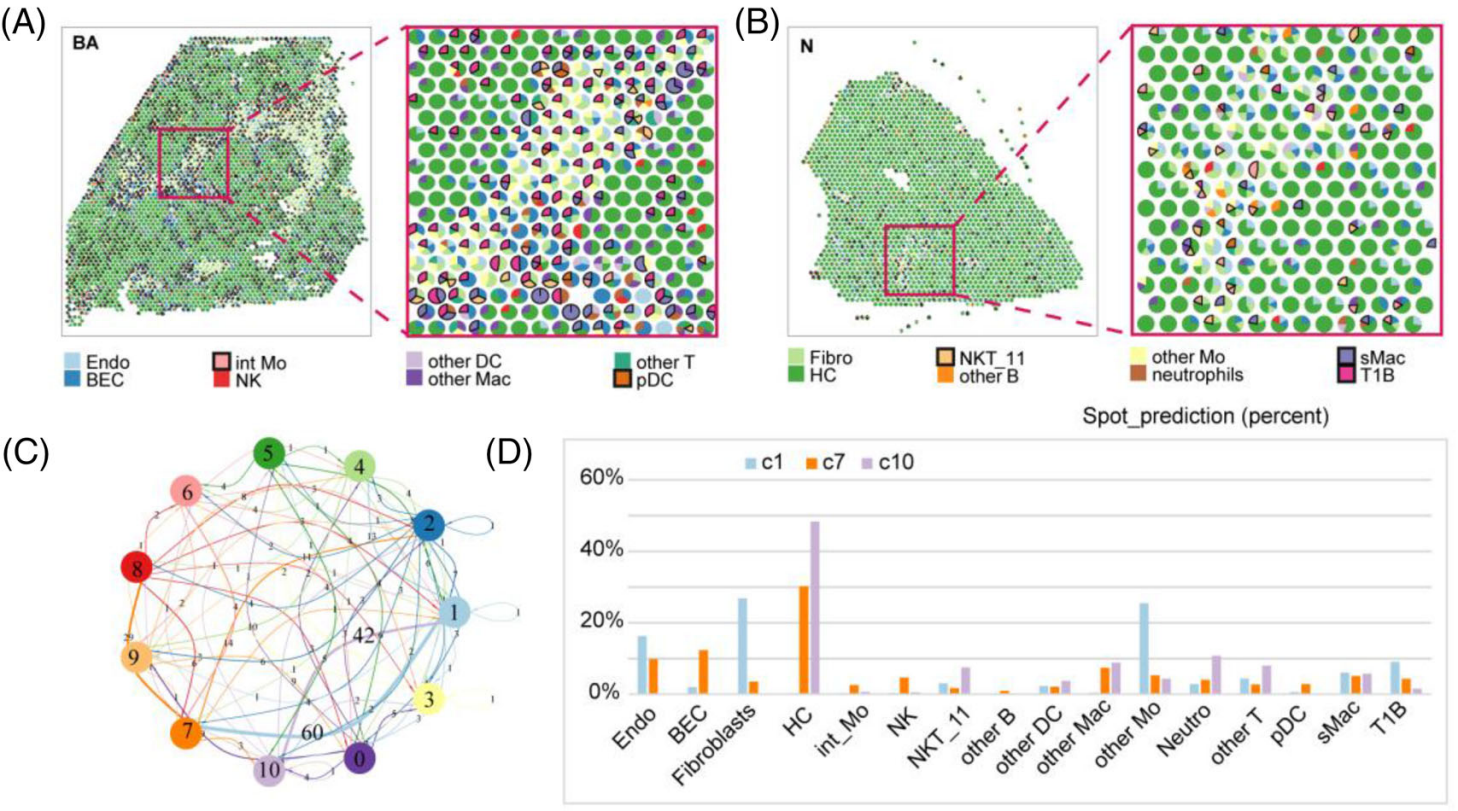
Deconvolution of ST data based on scRNA‐seq data. (A) Spot‐prediction pie chart of BA ST data: each small pie chart represents a spot, filled with colours representing different cell types. (B) Spot‐prediction pie chart of control ST data: each small pie chart represents a spot, filled with colours representing different cell types. (C) Cell–cell interactions: the thickness of the line represents the number of interactions. (D) Spot prediction histogram of clusters 1, 7 and 10 (c1, c7, c10)

SPOTlight was performed to further visualize the locations of the fibrosis‐related immune cells described above and to evaluate their fractions in BA and control sections. In line with the distribution of the HP zone and fibrotic niche/portal zone, we identified a striking segmentation of fibrosis‐related immune cells in the BA section (Figure 11A,B). We then computed cell–cell interaction networks in the BA section using Giotto. The results showed that clusters 1, 7 and 10 had the most interactions (Figure 11C; Appendices S8 and S9). Notably, 21/60 of the interactions were ‘FGF_FGFR’. According to their cellular composition, clusters 7 and 10 mainly consisted of hepatocytes and cluster 1 mainly consisted of int and fibroblasts (Figure 11D, Appendix S10).

### 3.9 Validation analysis by deconvolution of BA microarray data

**FIGURE 12:**
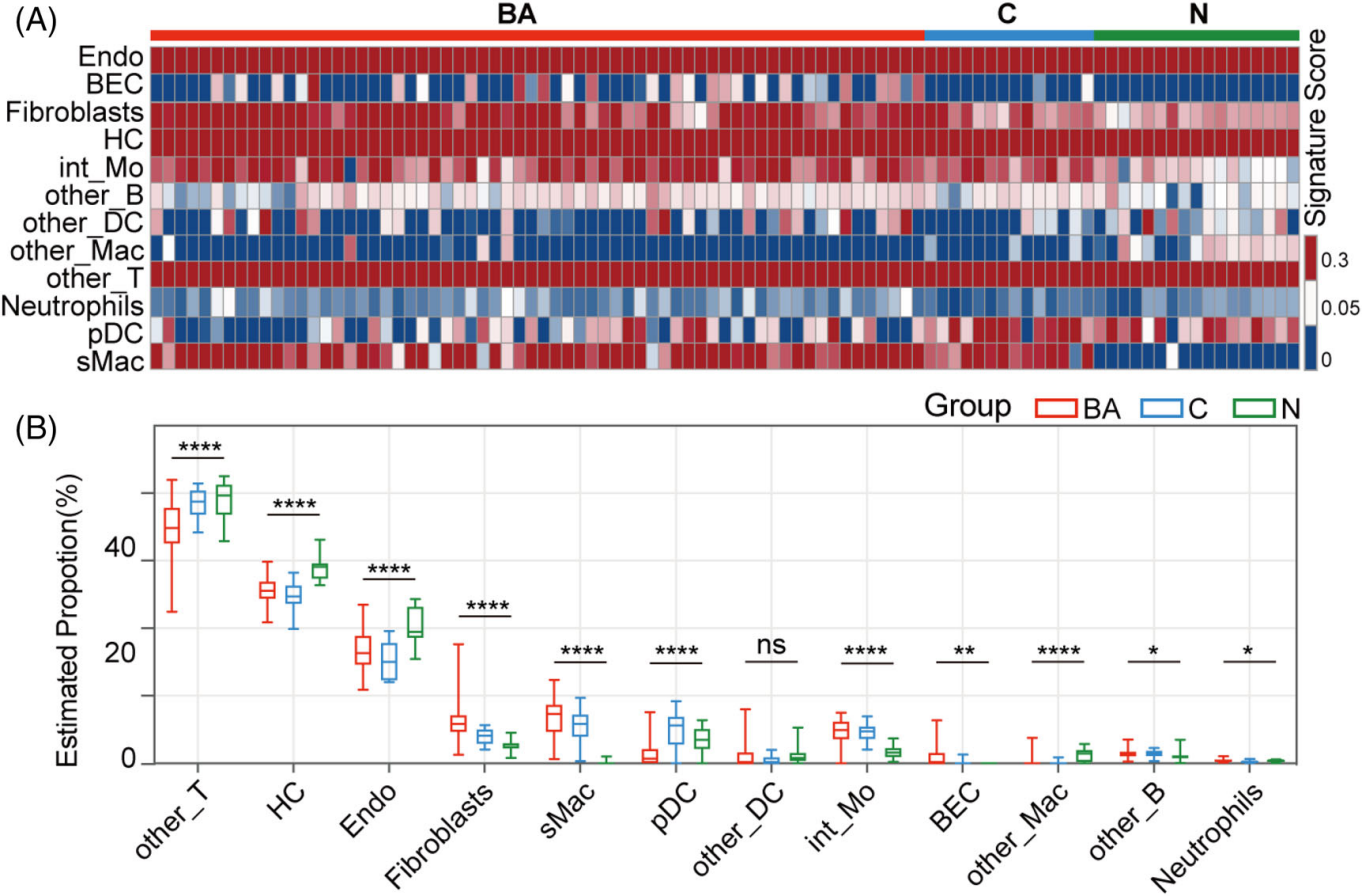
Deconvolution of BA microarray data based on scRNA‐seq data. (A) Signature score heatmap: signature score of BA microarray data calculated in CIBERSORTx based on our BA scRNA‐seq data. (B) Boxplot: boxplot of estimated proportions of different cell types of BA microarray data calculated in CIBERSORTx based on our BA scRNA‐seq data. The colour indicates the group (*p* < .0001, ****; *p* < .01, **; *p* < .05, **; ns, no significance, *t*‐test).

To validate the proportions of fibrosis‐related immune cells in a larger cohort, CIBERSORTx was applied to deconvolute a BA microarray data (including 64 BA infants, 14 intrahepatic cholestasis controls and seven normal controls)^14^ based on our scRNA‐seq data. In terms of BA, intrahepatic cholestasis controls and normal controls, ‘intermediate’ CD14^++^CD16^+^ Mo accounted for 4.81%, 4.67% and 1.63%, respectively; and sMac accounted for 6.72%, 5.53% and 0.06%, respectively (Figure 12; *p* < .0001; Appendix S11), and pDC accounted for 1.43%, 4.94% and 3.55%, respectively (Figure 12; *p* < .0001; Appendix S11). ‘FCN3^+^’ neutrophils, NKT, T1B and other Mo were not identified in the results, possibly because of the low cell numbers or other unknown reasons.

## 4 DISCUSSION

BA is a severe inflammatory and fibrosing cholangiopathy of neonates. Abnormal rapid liver fibrosis is the primary reason for the poor prognosis of this disease. In this study, we used two recently developed research methods (scRNA‐seq and ST) to establish a complete fibrosis‐related immune atlas of BA. First, we drew a complete immune microenvironment atlas of BA at single‐cell resolution. Second, we combined the spatial expression of each subgroup to identify subtypes that specifically clustered in the fibrotic niche, including pDC, ‘intermediate’ CD14^++^CD16^+^ Mo, sMac, NKT cells (NK & T, cluster 11), T1B and FCN3^+^ neutrophils. Further GO functional analysis showed that these subgroups were enriched for biological functions, including ‘positive regulation of SMC and fibroblast proliferation’ and ‘positive regulation of/response to VEGFR/VEGF/EGFR/FGF’. The differences in expression and spatial position of molecules in signaling ‘positive regulation of SMC and fibroblast proliferation’ were then explored (we chose these two factors because fibroblasts are considered important in liver fibrosis). We found that the expression of some genes in these two pathways, including *CYBA*, *AIF1*, *CD74* and *S100A6*, was increased in BA, especially in the fibrotic niche. Third, we found that many cell subgroups were enriched in the GO function ‘neutrophil chemotaxis’ when exploring the function of each immune subgroup. Further interaction analysis showed that neutrophils and other immune cells had extensive ‘RPS19‐C5AR1’ communications, and previous studies showed that C5AR1 is closely related to liver fibrosis.^24^ Moreover, interaction analysis of ST data showed that ‘FGF_FGFR’ pairs were extensive at the fibrotic niche and its boundary.

The newly discovered fibrosis‐related immune subgroups in this study (except that sMac, which was previously reported as related to fibrosis in a study of adult cirrhotic livers^10^) are novel findings. Ramachandran et al. performed functional studies to show that the ‘TREM2^+^CD9^+^’ sMac promoted liver fibrosis through TNFSF12‐TNFRSF12A and PDGFβ‐PDGFR α. In our study, we used enrichment analysis to identify multiple pathways that promoted fibrosis, and we discovered some highly enriched receptor ligand pairs, including ‘CD74_COPA/MIF/APP’ and ‘TNFRSF1A/B_GRN’, which were different from those identified in the previous studies. This suggests that there may be different specific key mechanisms of liver fibrosis in different diseases, given that the speed of liver fibrosis in children with BA is much faster than that in cholestatic liver fibrosis in adults and children with other conditions. In addition, previous studies of DCs,^4^, ^26^ NK cells,^5^, ^27^ T cells^28^, ^29^ and B cells^30^ in BA have mostly focused on the role of these cells in epithelial injury or immune immaturity/dysregulation, but not fibrosis. The present study reports spatial expression and functional analysis of each cell subgroup for the first time, showing that these specific cell subtypes may be directly related to liver fibrosis. We hope this will provide some new ideas and directions for future research on liver fibrosis in BA.

From the perspective of molecular mechanisms, liver fibrosis is characterized by dysregulation of physiological remodelling, activation of myofibroblasts and formation of a fibrous scar. The proposed sources of myofibroblasts in liver fibrosis include mesenchymal stromal cells, fibrocytes, mesothelial cells, portal fibroblasts, SMCs and hepatic stellate cells.^31^, ^32^ Here, subtypes of immune cells inhabiting the liver fibrotic niche all displayed functional enrichment of the GO term ‘positive regulation of SMC/fibroblast proliferation’. Meanwhile, numerous growth factors (especially VEGF, EGF and FGF^33^) and key genes (especially *CYBA*,^34^ *AIF1*,^35^ *CD74*,^36^ *S100A6* ^37^ and *C5AR1* ^24^) were reported to promote fibrosis. Similarly, the related functions ‘positive regulation of/response to VEGFR/VEGF/EGFR/FGF’ were also enriched in these subgroups. Therefore, the above studies and functional analysis results provide strong evidence of the role of these cell subpopulations in promoting liver fibrosis.

After an exploratory study using the scRNA‐seq data for a small collection of biospecimens, we validated the proportions of fibrosis‐related immune cells in a larger cohort using CIBERSORTx. However, further experimental validation and support with larger sample sizes are still needed in the future.

In summary, our study reveals the molecular, cellular and spatial immune microenvironment of the fibrotic niche of BA for the first time, providing further insight into the relationship between the immune microenvironment and liver fibrosis in BA.

## CONFLICT OF INTEREST

The authors declare that they have no conflicts of interest.

## Supporting information

Table S1: The clinical basic information of BA and control patients

Figure S2: The quality control map of scRNA‐seq data

Table S3: Canonical lineage markers of main cell lineages

Figure S4: Single R annotation of scRNA‐seq data

Table S5: Top 5 DEGs of main lineages of scRNA‐seq data

Appendix S6: Proportions of MP clusters of the BA and control group in scRNA‐seq data

Appendix S7: Biological processes (BP) of fibrosis‐related subclusters

Appendix S8: The number of interactions between clusters by Giotto

Appendix S9: The estimated cell interactions between clusters by Giotto

Appendix S10: The estimated cellular composition of clusters 1, 7 and 10 of ST data by SPOTlight

Appendix S11: The estimated proportions of fibrosis‐related immune cells in the GSE46960 cohorts
