# Supplementary figures and images for: Single‐cell and spatial transcriptomics reveal the fibrosis‐related immune landscape of biliary atresia
Source: Clin Transl Med. 2022 Nov 4;12(11):e1070. doi: 10.1002/ctm2.1070 (PMC9636046; doi:10.1002/ctm2.1070)

S2 Figure


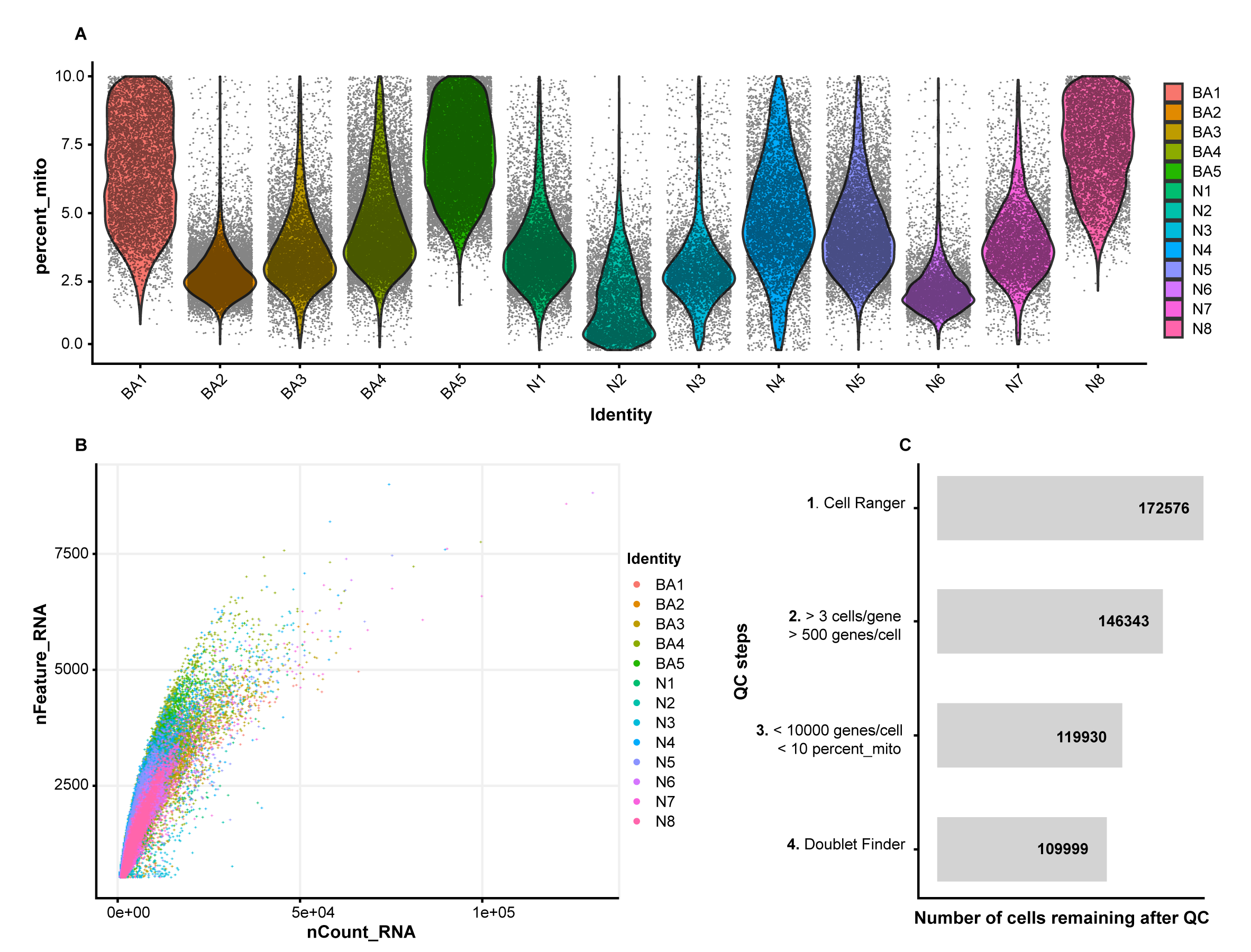

Supplement: Supplementary file 2 — Figure S2: The quality control map of scRNA‐seq data [file CTM2-12-e1070-s004.docx]

S4 Figure


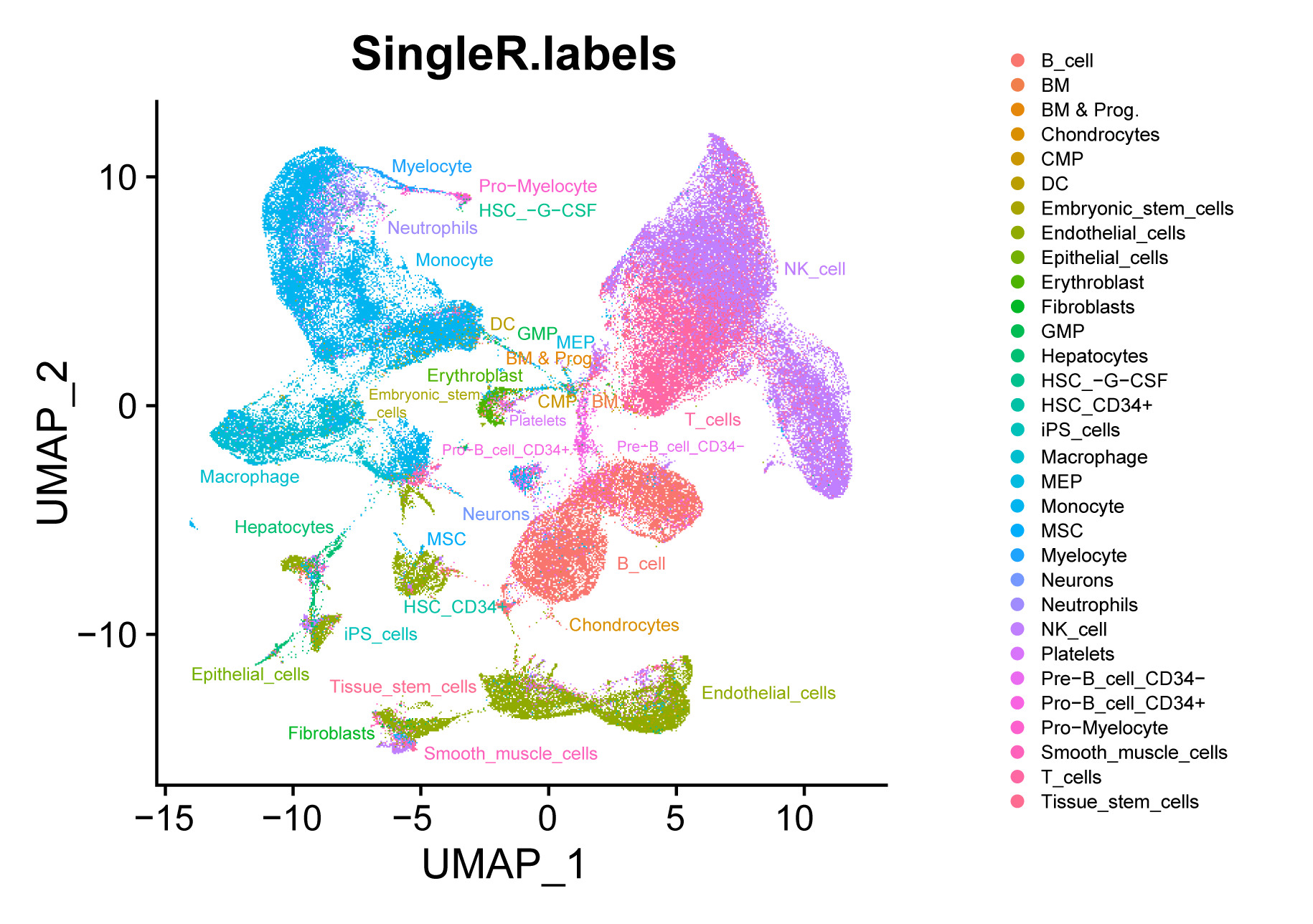

Supplement: Supplementary file 4 — Figure S4: Single R annotation of scRNA‐seq data [file CTM2-12-e1070-s003.docx]
